# ICES Data and Analytic Services: Eight Years Young.

**DOI:** 10.23889/ijpds.v7i3.2096

**Published:** 2022-08-25

**Authors:** Minnie M. Ho, Stefana Jovanovska, Jenna Novess, Dina Skvirsky, Refik Saskin, J. Charles Victor

**Affiliations:** 1 ICES

## Objectives

In March 2014, ICES launched Data & Analytic Services (DAS), expanding the access to ICES data and analytics beyond ICES scientists and analytic staff. In eight years, DAS has grown and evolved to increase high quality services offered to an expanding client base of external researchers.

## Approach

At the inception of DAS, two services were offered to public sector researchers: data access and analytics. Data access enabled researchers to analyze coded record-level data through a secure virtual environment. Analytics, conducted by DAS staff in ICES analytic environment, provided researchers with risk-cleared summary level reports. In response to growing demand from an increasingly diverse range of researchers, ICES engaged in extensive consultations with internal and external stakeholders to re-evaluate and operationalize new services. Compliance with contractual obligations and Ontario law, organizational capacity to scale up, alignment with ICES’ mission, vision and values, were cornerstones in establishing new offerings.

## Results

Analytic services became available to private sector researchers in June 2016. In March 2017, support for cohort and longitudinal follow-up studies became the newest service offering (researchers provided with a list of applicable individuals defined for the purposes of conducting publicly funded research). As more data assets become available to researchers, requests continue to increase in volume and complexity, particularly of projects seeking to import external data for linkage to ICES data. A second high performance computing virtual environment onboarded researchers September 2021 while the original analytic environment has undergone multiple upgrades, and will soon be fully refreshed. Regular solicitation of feedback has enabled DAS to increase staffing and diversify resources available which improves the client experience at all stages.

## Conclusion

Since its inception, DAS has expanded from five to thirty personnel, grown and diversified its new and returning client base and has responded to demand for new services. DAS continues to provide high quality services which enable impactful research and is responsive to new opportunities for collaboration and service provision.

